# Infected erythrocyte-derived extracellular vesicles alter vascular function via regulatory Ago2-miRNA complexes in malaria

**DOI:** 10.1038/ncomms12727

**Published:** 2016-10-10

**Authors:** Pierre-Yves Mantel, Daisy Hjelmqvist, Michael Walch, Solange Kharoubi-Hess, Sandra Nilsson, Deepali Ravel, Marina Ribeiro, Christof Grüring, Siyuan Ma, Prasad Padmanabhan, Alexander Trachtenberg, Johan Ankarklev, Nicolas M. Brancucci, Curtis Huttenhower, Manoj T. Duraisingh, Ionita Ghiran, Winston P. Kuo, Luis Filgueira, Roberta Martinelli, Matthias Marti

**Affiliations:** 1 Department of Immunology and Infectious Diseases, Harvard T.H. Chan School of Public Health, Boston, Massachusetts 02115, USA; 2 Department of Medicine, Unit of Anatomy, University of Fribourg, 1700 Fribourg, Switzerland; 3 Department of Biostatistics, Harvard T.H. Chan School of Public Health, Boston, Massachusetts 02115, USA; 4 Harvard Catalyst Laboratory for Innovative Translational Technologies, Harvard Medical School, Boston, Massachusetts 02115, USA; 5 Wellcome Trust Center for Molecular Parasitology, University of Glasgow, Glasgow G12 8TA, UK; 6 Division of Allergy and Infection, Beth Israel Deaconess Medical Center, Boston, Massachusetts 02115, USA; 7 Predicine, Inc., Hayward, California 94545, USA; 8 Center for Vascular Biology Research, Beth Israel Deaconess Medical Center, Boston, Massachusetts 02115, USA

## Abstract

Malaria remains one of the greatest public health challenges worldwide, particularly in sub-Saharan Africa. The clinical outcome of individuals infected with *Plasmodium falciparum* parasites depends on many factors including host systemic inflammatory responses, parasite sequestration in tissues and vascular dysfunction. Production of pro-inflammatory cytokines and chemokines promotes endothelial activation as well as recruitment and infiltration of inflammatory cells, which in turn triggers further endothelial cell activation and parasite sequestration. Inflammatory responses are triggered in part by bioactive parasite products such as hemozoin and infected red blood cell-derived extracellular vesicles (iRBC-derived EVs). Here we demonstrate that such EVs contain functional miRNA-Argonaute 2 complexes that are derived from the host RBC. Moreover, we show that EVs are efficiently internalized by endothelial cells, where the miRNA-Argonaute 2 complexes modulate target gene expression and barrier properties. Altogether, these findings provide a mechanistic link between EVs and vascular dysfunction during malaria infection.

## Abstract (web summary)

Inflammatory response to malaria pathogen *Plasmodium falciparum* can be triggered by infected red blood cell-derived extracellular vesicles (EVs). This study shows these EVs contain functional microRNA-Argonaute 2 complex that modulates gene expression and alter vascular barrier properties.

## Graphical abstract

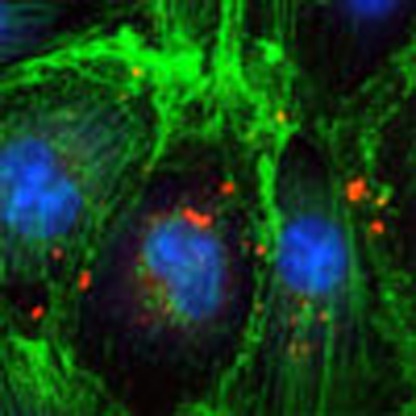

Despite continuing efforts, malaria remains a major public health threat worldwide. Although the majority of infections remain asymptomatic or uncomplicated, a small proportion of infected people develops severe symptoms and may die. The most virulent malaria parasite, *P. falciparum* causes ∼650,000 deaths annually, most of them amongst children below the age of 5 in sub-Saharan Africa^1^. The factors that regulate the transition from an uncomplicated disease to serious conditions including cerebral malaria (CM) and severe malarial anaemia are poorly understood. Even when treated CM has a poor prognosis with fatality rates of 30–50%, and survivors often suffer from neurological complications.

The pathology of *P. falciparum* malaria is related to the capability of parasite-infected red blood cells (iRBCs) to sequester in deep tissues by adherence to the microvasculature. For this purpose the parasite expresses a variant surface antigen, *P. falciparum* erythrocyte membrane protein 1 (PfEMP1). Each *P. falciparum* parasite genome encodes about 60 PfEMP1 variants that enable the parasite to attach iRBCs to different host receptors on the vascular lining of various tissues, including brain and placenta^2^. Sequestration prevents splenic clearance of mature iRBCs and contributes to pathology by reducing blood flow and promoting inflammation in the capillaries. Inflammatory cytokines can induce receptor expression on the surface of the endothelium, thereby enhancing iRBC binding. In addition, adhesion of iRBCs to the endothelium can increase vascular permeability and apoptosis of the endothelial cells^3^. Indeed, vascular dysfunction is a common feature of cerebral malaria through destruction of the blood–brain barrier and the formation of haemorrhages^4^,^5^,^6^. Interestingly, *in vitro* experiments have demonstrated that increased vascular permeability does not only require direct iRBC interactions but can also be caused by soluble factors released by iRBCs^7^. Transfer of released malaria antigens to endothelial cells has also been observed in autopsy studies in the context of acute inflammatory lesions in cerebral tissue^5^,^8^. Likewise tissue surveys in autopsy studies have detected parasite markers in endothelial cells of multiple tissues^9^. Antigen transfer from iRBCs to brain endothelial cells appears to occur via membranous structures during transient iRBC—endothelial cell interactions^7^, a process reminiscent of trogocytosis.

Release of membranous material in the form of extracellular vesicles (EVs) from iRBCs during parasite development has initially been described in the rodent malaria model and more recently in *P. falciparum*^10^,^11^,^12^,^13^. EVs are small vesicles released by almost all eukaryotic cell types. EVs derived from iRBCs are increased in serum of malaria patients, in particular during severe disease^14^. EVs can activate cells of the innate immune system in the rodent malaria system and in human malaria^11^,^13^. We have recently demonstrated that EVs can also facilitate cellular communication between iRBCs by transferring signalling molecules from a donor to a recipient iRBC. Moreover this communication pathway is linked to the formation of malaria transmission stages^11^ and capable of transferring (episomal) DNA^10^.

Compositional analysis of EVs derived from iRBCs identified a specific set of RBC and parasite proteins as well as RNA species including small RNA species^11^. In other systems, EVs have been shown to contain nucleic acids, in particular messenger RNA (mRNA) and microRNA (miRNA) that can be transferred to and function in recipient cells. For example, Epstein-Barr virus induces secretion of EVs that contain viral miRNAs from infected cells, and these secreted viral miRNAs can downregulate cytokine expression in uninfected monocyte-derived dendritic cells^15^. Similarly, hepatitis C virus induces secretion of exosomes from infected hepatocytes that are also able to transmit the virus to naive cells^16^. Importantly, extracellular miRNAs in complex with proteins and lipids are very stable and protected from degradation by RNAses and are therefore particularly well suited to transduce signals^17^. miRNAs are 19–24-nucleotide noncoding RNAs that are derived from larger primary transcripts encoded in the genome. Following initial processing by the RNases Drosha and DGCR8 in the nucleus, the pre-miRNA is exported to the cytoplasm where it is processed to a miRNA duplex by another RNase termed Dicer^18^. One of the RNA strands is loaded and incorporated into the effector protein Argonaute 2 (Ago2) to form the RNA-induced silencing complex (RISC)^19^. The sequence-specific miRNA guides the RISC complex either to target sites in coding regions of target mRNAs for endonucleolytic cleavage or to the 3′-untranslated regions (UTRs) leading to translational repression^20^. The latter can occur through a variety of reported mechanisms including decreased mRNA stability due to deadenylation and uncapping, or via direct inhibition of translation^21^.

In this study, we tested the hypothesis that iRBC-derived EVs contain miRNAs that can modulate target genes in recipient cells. We identified multiple miRNA species in EVs, and we demonstrated that they are bound to Ago2 and form functional complexes. Furthermore, we observed transfer of iRBC-derived EVs into endothelial cells, repression of miRNA target genes and alteration of endothelial barrier properties.

## Results

### iRBC-derived EVs contain a subset of human miRNAs

**Figure 1:**
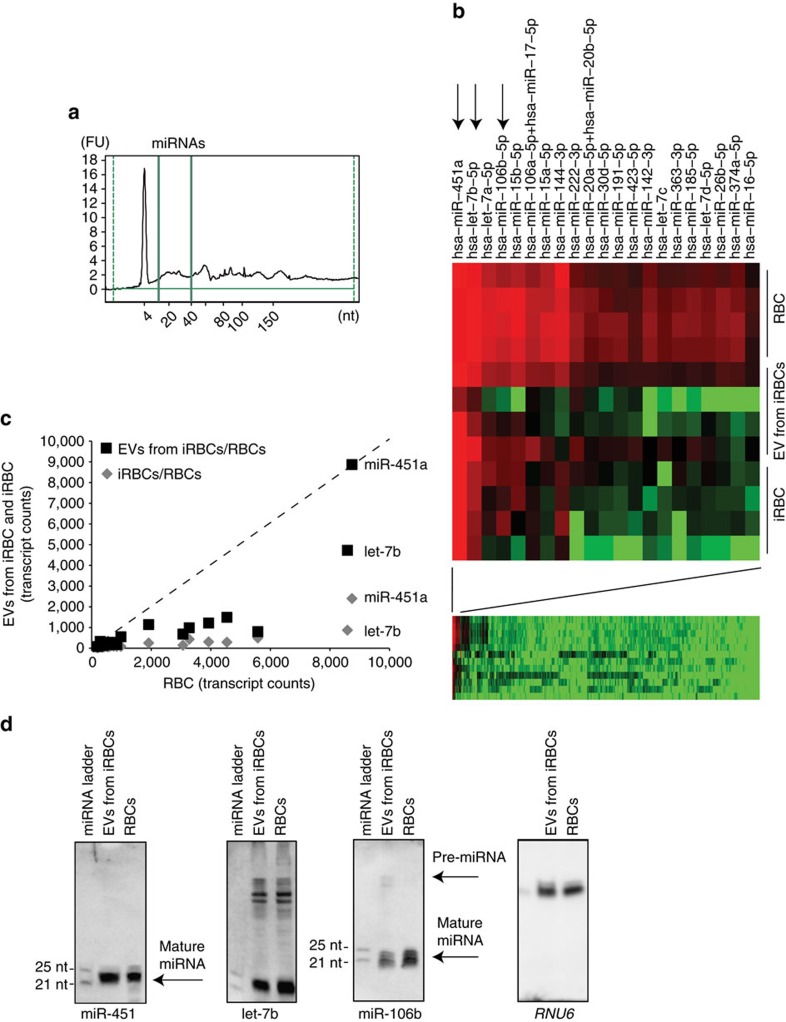
Detection of human miRNA species in EVs. (**a**) EVs derived from iRBCs contain small RNAs. RNA samples were prepared from EVs and analysed by Agilent Bioanalyzer Small RNA Chip. Small RNA species of up to 150 nucleotides are detected, including species between 20 to 25 nucleotides (range marked by green lines). FU, fluorescence units. (**b**) miRNA profiling of iRBCs, EVs from iRBCs and RBCs by nanostring. miRNA preparations from four experiments were analysed using a Nanostring miRNA array containing 800 human miRNA species. Normalized data from the entire array are shown in the left panel. Twenty-one miRNAs are expressed in RBCs, of which a subset is also present in iRBCs and EVs from iRBCs (right). These are (in order of expression levels) miR-451a, let-7b, let-7a, miR-106b and miR-15b. Those that are further characterized in this study are marked with black arrows. (**c**) Differential abundance of miRNA species in RBCs, EVs from iRBCs and RBCs. The normalized Nanostring data from **b** are represented as correlations between the mean expression across four experiments of RBC and the mean expression of either iRBCs (black boxes) or EVs from iRBCs (grey diamonds). miR-451a and let-7b show similar abundance across all three populations, while the other miRNA species are greatly reduced in iRBCs and EVs from iRBCs. (**d**) Detection of miRNAs by northern blot. RNA samples from RBCs and EVs from iRBCs are hybridized with probes specific for miR-451a, let-7b and miR-106b. The northern blot shows specific bands at the expected size of 22 nucleotides for miR-451a and let-7b, and of 21 nucleotides for miR-106b. In addition unprocessed Pre-miRNA species at the expected size of 70 nucleotides are detectable for let-7b and miR-106b. Shown is also a blot for U6 small nuclear RNA (*RNU6*) as a control for RBC-derived non-miRNA RNA species^23^. Samples were normalized using equal RNA input.

We have previously developed a protocol for purification of iRBC-derived EVs, and we have demonstrated the presence of specific parasite and host proteins in purified EVs, including human Ago2. Initial experiments also demonstrated that EVs from iRBCs contain RNA, although the nature of these RNA species remained unclear^11^. To further confirm these initial findings and investigate the composition of nucleic acids in EVs, we prepared purified EVs from iRBC cultures and analysed the size distribution of RNA by the Agilent 2100 bioanalyzer (Fig. 1a). Starting with 1 mg of purified EVs we extracted total RNA. For this purpose EVs were pretreated with RNase A before lysis in order to remove potential contaminating free RNA bound to EVs or co-purified during the isolation process. We also excluded possible serum contamination by pre-clearing the serum of host EVs with ultracentrifugation before culturing the parasites. Analysis by bioanalyser failed to detect RNA species above 150 nucleotides in size, including the diagnostic ribosomal RNA bands (Supplementary Fig. 1a). In contrast we detected small RNA species in the range of 21–25 nucleotides suggesting the presence of mature miRNAs (Fig. 1a). Since *Plasmodium* parasites lack functional RNA interference machinery capable of producing miRNAs^22^, we hypothesized that these miRNA were host-derived. Indeed it has previously been shown that human RBCs express a small set of miRNA species during maturation, with various roles during RBC development^23^. To profile the composition of human miRNAs in EVs from iRBCs, we used a nanostring expression array comprising probes for all 800 human miRNA species. Specifically, we compared the composition and amount of human miRNAs from uninfected RBCs, infected RBCs and EVs. As expected, we detected RBC-specific miRNAs in all four preparations with a total of 21 miRNAs specifically present in RBCs and a small subset of those in iRBCs and EVs from iRBCs (Fig. 1b and Supplementary Data 1). However, except for miR-451a and let-7b, all the miRNA species were depleted in iRBC and EV samples as compared with uninfected RBCs (Fig. 1c). Notably, miR-451a is the most abundant miRNA in RBCs and plays an important role in erythroid homoeostasis during RBC development^24^. To independently confirm presence of mature miRNA species in RBCs, iRBCs and EVs, we performed northern blots for miR-451a, let-7b, *RNU6* and miR-106b (Fig. 1d). All four miRNA species were detected in RBCs and EVs prepared from iRBCs culture supernatants. Altogether these data demonstrate the presence of a subset of RBC-specific miRNAs, notably the RBC-specific miR-451a species, in EVs and iRBCs.

### Mature RBCs and iRBCs contain human Ago2 protein

**Figure 2:**
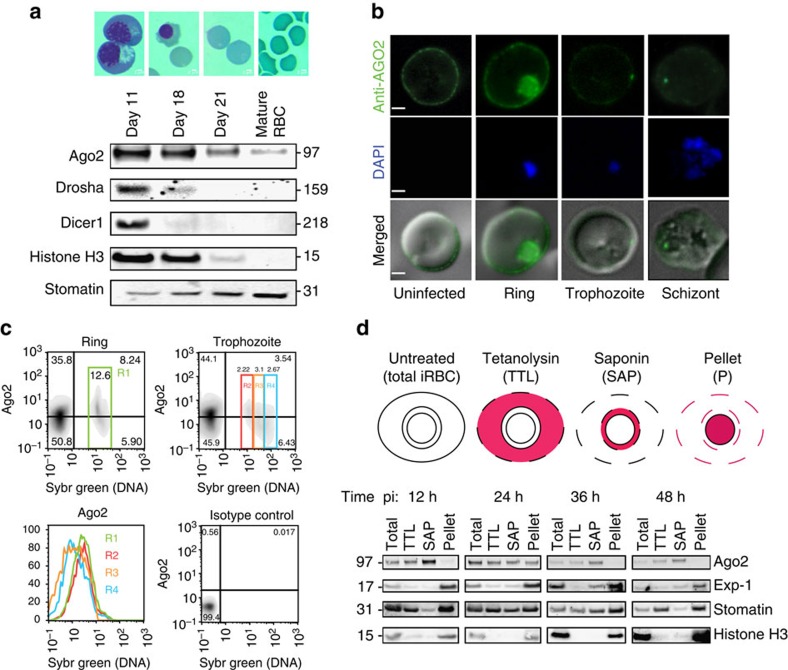
Detection of human Ago2 in RBCs and iRBCs. (**a**) Expression of RNAi machinery during RBC development. Cell lysates from different developmental stages of erythropoiesis were prepared (day 11: basophilic and polychromatic erythroblasts, day 18: orthochromatic erythroblasts and reticulocytes, day 21: reticulocytes and normocytes; representative images are shown). Ago2 is detectable in mature RBCs while dicer, drosha (and human histone H3) are not present. Stomatin is present in all preparations. (**b**) IFA of Ago2 in RBCs and iRBCs. Ago2 is localized to the RBC periphery in uninfected and infected RBCs, however, labelling is reduced in later parasite stages. Notably, ring stage parasites also show Ago2 accumulation in the parasite. Scale bar; 1 μm. (**c**) Flow cytometry analysis of Ago2 labelling in RBCs and iRBCs. Uninfected RBCs and iRBCs were gated based on SYBR staining (nuclear content, *n*) and Ago2 labelling. Young iRBCs (rings, and trophozoites with *n*=1) show the highest Ago2 labelling, confirming IFA data. (**d**) Sequential fractionation of purified infected parasites analysed by western blot. iRBC samples were collected at four time points post invasion, separated from uninfected RBCs and fractionated. The host cytosol was released with 1 HU of tetanolysin (TTL) and subsequently the PV contents were released with 0.035% of saponin (SAP). The pellet contains parasite material and host membranes while the TTL supernatant contains RBC cytosol and the SAP supernatant contains soluble parasitophorous vacuole material. The parasitophorous vacuole membrane marker PfExp-1 and parasite nuclear histone H3 are detectable in the pellet fraction across the asexual parasite cycle. Ago2 is present in cytosol (SAP, TTL) across the cycle but only present in the pellet fraction in early parasite stages, supporting the dynamic distribution observed by IFA. Host stomatin is present in cytosol (SAp, TTL) and membranes (pellet) throughout the cycle. Data are representative of three independent experiments. pi: post invasion.

There is increasing evidence for the EV-mediated transfer of miRNAs between cells. Although generally subject to degradation by nucleases, mature miRNAs are protected and functional when bound to Ago2 (ref. 25). We therefore hypothesized that the miRNA detected in iRBCs and EVs may be bound to Ago2. Functionality of RNAi has been described in erythroid cells^26^, but there is no evidence so far that the machinery remains functional once erythroid cells mature to normocytes (that is, mature RBCs). To investigate whether functional miRNAs may be present in mature RBCs, we first monitored expression of components of the RNAi machinery during *in vitro* RBC maturation from hematopoietic stem cells to normocytes^27^. We detected Ago2 in mature RBCs, while Drosha and Dicer were only detectable in erythroid cells, before their enucleation and maturation into reticulocytes (Fig. 2a). To determine the localization of Ago2 in mature RBCs, we performed immunofluorescence assays (IFA) and flow cytometry using specific antibodies to human Ago2. In normocytes, Ago2 was primarily localized to the cell periphery, close to the RBC membrane (Fig. 2b). Analysis of infected RBCs demonstrated a similar localization pattern in RBCs containing young ring stage parasites (up to ∼20 h post invasion). Later during development, however, the signal was progressively lost (Fig. 2b,c). We have previously observed a similar dynamic in subcellular localization for the RBC lipid raft protein stomatin, with stomatin disappearance coinciding with the peak of EV release from iRBCs during late schizont maturation^11^. We further characterized the subcellular localization of Ago2 during iRBC development by sequential permeabilization using the pore-forming agents tetanolysin and saponin. Tetanolysin forms pores in the RBC membrane but leaves the internal membranes intact, while saponin also permeabilizes the parasitophorous vacuole membrane while leaving the parasite intact^28^(Fig. 2d). Sequential treatment of iRBCs with tetanolysin and saponin results in a RBC cytosol, a parasitophorous vacuole and a parasite and host membrane fraction (pellet). Western blot analysis demonstrated Ago2 presence in the soluble fractions representing RBC cytosol and parasitophorous vacuole. During the first 24 h of parasite development, the protein was also present in the pellet fraction, confirming the observed IFA localization in the parasite and at peripheral RBC membranes and/or cytoskeleton. At later time points, the protein could not be detected in the pellet anymore, suggesting relocalization or secretion, possibly via EVs.

### Ago2 and miRNA are found inside EVs

**Figure 3:**
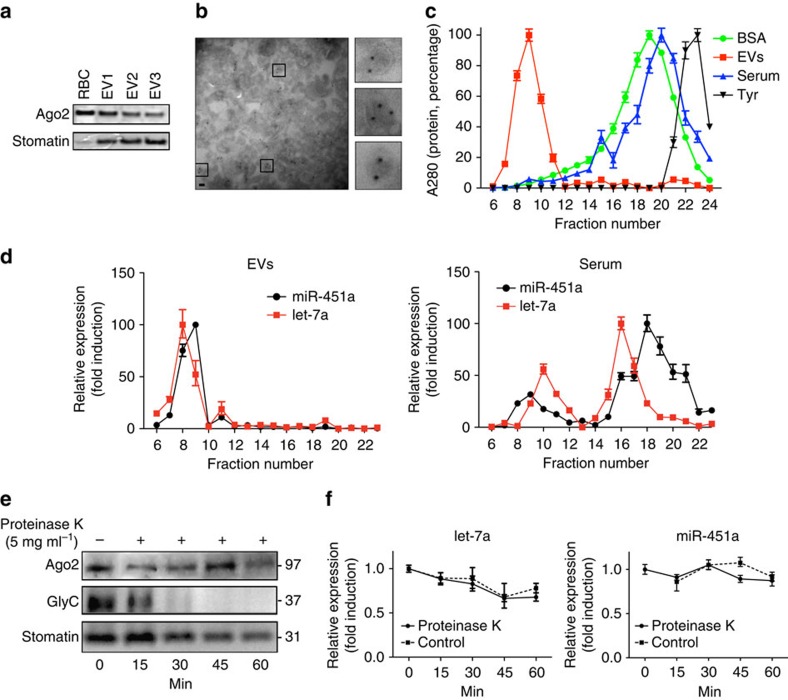
Ago2 and miRNA are present within purified EVs. (**a**) Expression of Ago2 and stomatin in multiple EV preparations was probed by western blot. (**b**) Immuno electron microscopy. EVs were prepared and processed as described in materials and methods for labelling with human Ago2 antibody. The field of view shows multiple EVs with internal immunogold labelling, demonstrating that Ago2 is present in EVs rather than associated by surface attachment. Inserts: three representative EVs in higher magnification (scale bar, 100 nm). (**c**) Elution profile of purified EVs, human serum, BSA and tyrosine (Tyr) after size-exclusion separation. Protein abundance was determined by absorbance at 280 nm. Points represent the mean±s.d. of three experiments performed in triplicate. (**d**) Fractions from purified EVs (left) and serum (right) were assayed for miR451a (black) and let-7a (red) using absolute quantification by TaqMan qPCR. The mean±s.d. of one representative experiment is shown (*n*=2). (**e**,**f**) The Ago2-miRNA complexes are protected from protease K digestion. (**e**) EVs were treated with proteinase K (5 mg ml^−1^, or untreated control) at 55 °C. At times indicated, aliquots were removed and assessed for Ago2, stomatin and Glycophorin C expression by western Blot. In contrast to Glycophorin C, Ago2 and stomatin were protected from digestion by proteinase K. (**f**) Untreated (open symbols) or treated samples (close symbols) were also analysed for miR-451a and let-7a expression by qPCR. Points represent miRNA copies detected at each time relative to the control sample at time point 0. The mean±s.d. of one representative experiment is shown (*n*=3). qPCR, quantitative PCR.

Indeed, we detected Ago2 (and stomatin) in EV preparations from iRBCs by western blot analysis (Fig. 3a), corroborating our previous proteomics data^11^. We independently confirmed that Ago2 is present within EVs by immune EM with specific anti-Ago2 antibodies using purified EVs (Fig. 3b). Previous work has demonstrated that most of the miRNAs found in the plasma of healthy donors are mainly associated with proteolipid complexes and not with EVs^29^. To exclude the possibility that Ago2 and miRNA molecules originate from protein–lipid complexes present in the serum, we used two complementary approaches, as recently described^29^. First, we performed size exclusion chromatography and demonstrated that human serum indeed contains a subpopulation of miRNAs (and proteins) that elute in the same fractions as those from EV preparations. Importantly, EV preparations do not elute miRNA or protein in the major serum fractions, demonstrating that our EV samples are devoid of serum contaminants (Fig. 3c,d). Second, Proteinase K protection assays with EV preparations demonstrated that Ago2 and miRNA molecules (and stomatin control) were protease resistant while the surface exposed Glycophorin C was sensitive (Fig. 3e,f), as we have shown previously^11^. Altogether these experiments demonstrate that Ago2 and miRNA molecules are present within EVs.

### miRNAs form a functional RISC complex with Ago2 in EVs

**Figure 4:**
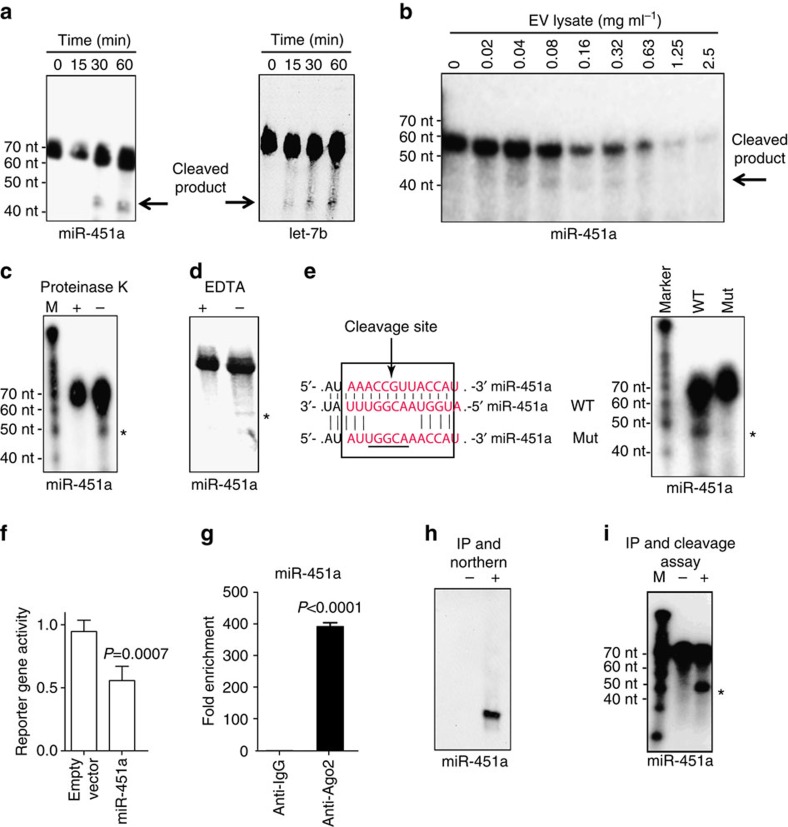
Characterization of a functional Ago2-miRNA complex in EVs that are functionally competent in gene silencing. (**a**,**b**). *In vitro* RISC activity assay. (**a**) EV lysates were incubated for 0, 15, 30 or 60 min in the presence of a ^32^P-labelled RNA sensor with binding site complementary to miR-451a (left) or let-7b (right). The cleavage was monitored by gel electrophoresis. (**b**) miR-451a sensor was incubated for 90 min with increasing amounts of EV lysate and emergence of processed product was monitored as above. The autoradiogram demonstrates emergence of product in a range between 0.04 and 0.32 mg EV lysate per ml. (**c**–**e**) Specificity of endonucleolytic RISC activity. Co-incubation of EVs and miR-451a probe with 1 mg ml^−1^ of proteinase K (**c**) or EDTA (**d**) inhibits the cleavage reaction, while a DMSO control remains unaffected. (**e**) Disruption of the miR-451a cleavage site on the probe (Mut) blocks processing, while the wild-type probe (WT) is efficiently cleaved. (**f**) 3′-UTR degradation activity. HEK293 cells transiently expressing a Renilla luciferase reporter gene with binding site complementary to miR-451a in the 3′-UTR of the reporter cassette were incubated with EVs derived from iRBCs for 48 h prior to luciferase activity measurements. Results are normalized based on constitutive Firefly luciferase activity, and expressed as mean (±s.e.m.) percentage of control (*n*=3 experiments). *P* versus control (Student's *t*-test). (**g**,**h**) Activity of purified Ago2-miRNA complex. (**g**) Quantification of miRNA by qRT-PCR. Protein extracts derived from purified EVs were subjected to immunoprecipitation (IP) using anti-Ago2 antibody, followed by miR-451a quantification using qRT-PCR. Results are normalized by the 2^-Ct^ method, using the *RNU6* gene as a reference and expressed as the enrichment versus the IgG1 negative control (mean ± s.e.m.; *n*=4 experiments). *P* versus control (Student's *t*-test). (**h**) Detection of miRNA by northern blot. Negative control represents IP using control IgG. (**i**) Activity of Ago2-miRNA complex. The precipitated complex was incubated with a miR-451a probe and cleavage confirmed, while the negative control shows no processing. ‘ * ‘ Indicates the expected cleavage product of the probes (46 nt).

miRNAs form a stable silencing complex with Ago2 (that is, the RISC complex) in order to be functional. RISC binds to the targeted mRNA through the seeding sequence of the miRNA. Having established presence of both specific miRNA species and Ago2 in EVs we next wanted to determine whether they formed a functional silencing complex. To test sequence-specific endonucleolytic activity of RISC we incubated purified EV lysates from infected RBCs with a radio-labelled RNA sensor probe containing a complementary sequence to either miR-451a or let-7b. This initial experiment demonstrated time-dependent cleavage activity starting at 30 min of incubation time (Fig. 4a). Next, we titrated the activity of EV lysate, demonstrating a window of specific cleavage of the miR-451a probe at 0.04 to 0.65 mg ml^−1^ of input lysate. At higher concentrations the probe is fully degraded, suggesting presence of nonspecific RNases in the EV lysate (Fig. 4b). Importantly, pre-treatment of EV lysate with either Proteinase K (to degrade proteins) or the Mg2+ chelator EDTA (to block enzymatic activity) inhibited the cleavage reaction, demonstrating requirement of active enzyme (Fig. 4c,d). We also demonstrated specificity of the endonucleolytic cleavage reaction, as a probe with mutated cleavage site was not processed when incubated with EV lysate (Fig. 4e). To further confirm the functionality of the RISC complex contained in EVs, we transiently transfected HEK293 cells with a reporter gene construct. In fact, the luciferase-based assay with miR-451a target sequence in the 3′-UTR of a reporter cassette demonstrated significant downregulation of the reporter gene activity in transfected HEK293 cells upon co-incubation with EVs (Fig. 4f). In contrast, we did not detect significant reporter downregulation when placing other miRNA target sequences in the 3′-UTR such as let-7b or miR-106a (Supplementary Fig. 1c). Indeed, the addition of a let-7b, miR-15a of miR-106a responsive element significantly decreased the activity of the luciferase reporter gene, even in the absence of EVs, indicating that HEK cells express a significant amount of those miRNAs (Supplementary Fig. 1b). These data demonstrated that EV-derived miR-451a complexes are capable of sequence-specific target gene degradation upon 3′-UTR association. To directly demonstrate physical interaction of Ago2 and miRNA in a complex in purified EVs, we performed immunoprecipitation with Ago2 antibodies (or an IgG isotype control). Quantitative real-time PCR (qRT-PCR) confirmed association of Ago2 with miR-451a (Fig. 4g), as well as with other miRNAs detected in EVs by our Nanostring analysis (Supplementary Fig. 1c). Furthermore mature miR-451a was detected by Northern blot only after immunoprecipitation with the Ago2 antibody, whereas it was not detectable in the isotype control immunoprecipitate (Fig. 4h). Cleavage assays using a radio-labelled miR-451a probe demonstrated functionality of the purified miR-451a–Ago2 complex (Fig. 4i). In summary, these results demonstrate that miRNAs in EVs are functional and require Ago2 association for activity.

### EVs from iRBCs are internalized by endothelial cells via endocytosis

**Figure 5:**
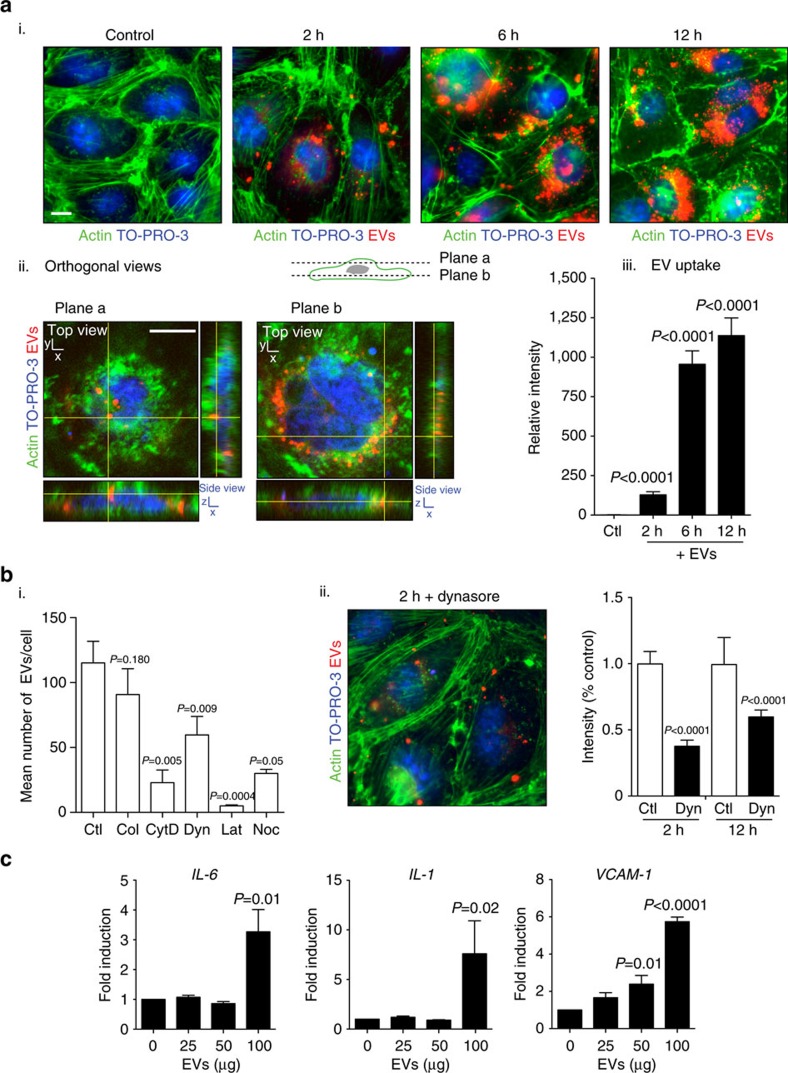
Uptake of EVs by endothelial cells. (**a**) Dynamics of EV internalization. Bone marrow endothelial cells (BMEC) were incubated with 100 μg of PKH26 fluorescently labelled EVs for 0, 2, 6 or 12 h. BMECs were stained for actin (phalloidin, green) and nuclei (TO-PRO-3, blue). (i) Following washing, the majority of internalized EVs were detected in a perinuclear region of the endothelial cell starting at 2 h post incubation. Scale bar, 10 μm. (ii) Orthogonal views of a representative cell after 12 h of EV uptake demonstrate that EVs are internalized. Scale bar, 7 μm. (iii) Internalized EVs were quantified by means of fluorescence intensity. Background signal was subtracted for every single image before obtaining the relative fluorescence per field. At least 15 images were collected per condition (mean ± s.e.m.; *n*=3 experiments). *P* versus control (Student's *t*-test). (**b**) Effect of inhibitors on EV uptake. Cells were co-incubated with inhibitors and EVs, uptake was quantified compared with control as in **a**. (i) Effect of known EV uptake inhibitors. Compounds were co-incubated for 2 h with EVs and the effect measured as mean number of EVs per cell. Ctl: control. Col: Colchicine (31 nM). CytD: Cytochalasin (600 nM). D. Dyn: Dynasore (80 μM). Lat: Latrunculin A (5 μM). Noc: Nocodazole (20 μM). (ii) Phenotypic effect of Dynasore inhibition. The drug was added at 80 μM and the effect measured (intensity of PKH26 staining) after 2 and 12 h of co-incubation with EVs. (mean ± s.e.m.; *n*=3 experiments). *P* versus control (Student's *t*-test). (**c**) Activation of cytokines and cell surface receptors upon EV uptake. Detection of *IL-1*, *IL-6* and *VCAM-1* by qRT-PCR in BMEC upon incubation with an increasing amount of EVs. qRT-PCR results are normalized by the 2^-Ct^ method, using EF-1 as a reference and expressed as mean and fold induction over control (mean ± s.e.m.; *n*=3 experiments), *P* versus control (Student's *t*-test).

Having established the presence of functional miRNA-Ago2 complexes in EVs, we wanted to determine their potential physiological role during malaria infection. We have previously shown EV internalization by iRBCs and macrophages^11^. To test whether EVs are internalized by endothelial cells and possibly contribute to the observed changes of endothelial function during malaria infection, we labelled purified EV preparations with a membrane dye and co-incubated them with a semi-immortalized human bone marrow-derived endothelial cell line (BMEC)^30^. We observed increasing uptake of labelled EVs over time, and confocal microscopy analysis demonstrated accumulation of labelled EVs in a perinuclear region in endothelial cells (Fig. 5a). Most experimental evidence suggests that EVs from various cell types are internalized into the cell via endocytosis^31^. Previous experiments in our laboratory and by Regev-Rudzki *et al*. have demonstrated that EV internalization by macrophages and by iRBCs is sensitive to the actin filament inhibitor Cytochalasin D (refs 10, 11). To test whether uptake of EVs from iRBCs into BMEC is via an endocytic pathway, we co-incubated endothelial cells either with EVs alone or together with a series of known endocytosis inhibitors that have previously been shown to block EV uptake^31^, and measured uptake over time. Specifically we tested the actin filament inhibitors Latrunculin A and Cytochalasin D, the microtubule inhibitors Nocodazole and Colchicine as well the Dynamin-2 inhibitor Dynasore^32^. Actin filament and microtubule inhibitors are known to block endocytosis^33^,^34^. Dynamin-2 is required both for Clathrin-mediated and Caveolin-dependent endocytosis and its inhibition through Dynasore can block EV uptake in other systems^35^. Except for Colchicine, all compounds had a significant effect on EV uptake (Fig. 5b), strongly suggesting that EV internalization by endothelial cells is indeed via endocytosis.

Similar to EV-mediated activation of macrophages^11^, we observed that EVs are able to induce pro-inflammatory cytokines such as interleukin-6 (IL-6) and interleukin-1 (IL-1) (Fig. 5c). Induction of these cytokines is known to impair endothelial barrier function^36^ and increase expression of adhesion molecules^37^ thereby contributing to vascular dysfunction by inducing excessive leukocyte adhesion and transmigration. In addition, EV internalization directly induces expression of surface receptor vascular cell adhesion protein 1 (VCAM-1) possibly exacerbating vascular dysfunction (Fig. 5c).

### EV-derived miRNA accumulates in endothelial cells

**Figure 6:**
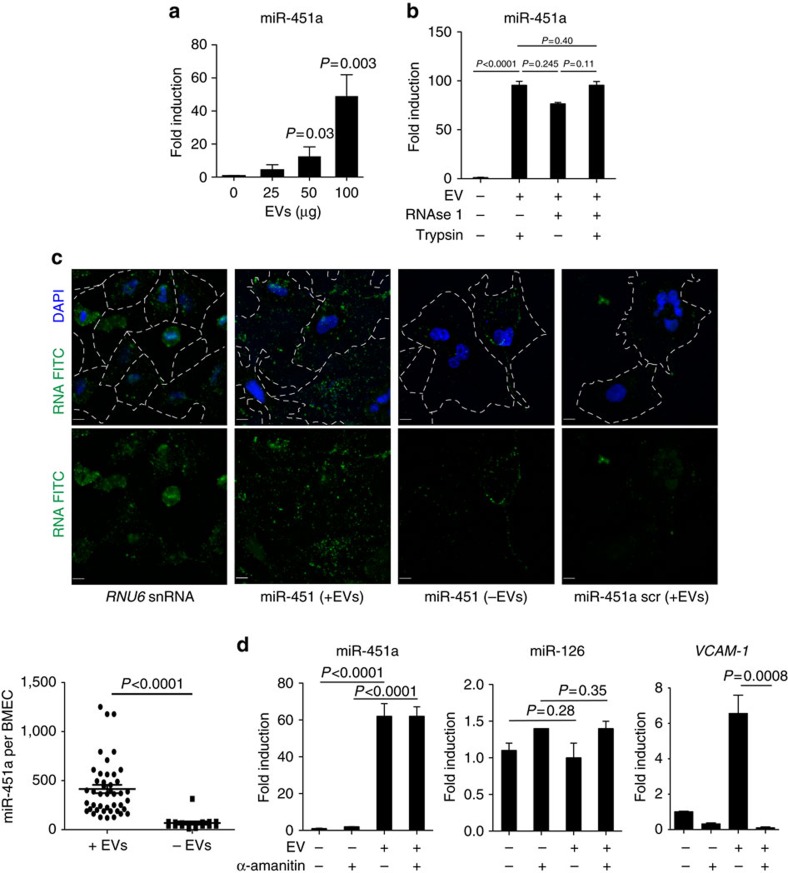
Accumulation of miRNA in BMEC upon EV uptake. (**a**) Detection of miRNA by qRT-PCR. miR-451a was quantified by qRT-PCR in BMEC upon incubation with an increasing amount of EVs during 24 h. (**b**) miRNA uptake is resistant to RNAse 1 and trypsin. BMEC was incubated with RNAse 1 and/or trypsin after EV uptake. miR-451a was quantified by qRT-PCR in endothelial cells. (**c**) Detection of miRNA by RNA FISH. miRNA transfer by EVs is measured by RNA FISH. miR-451a is only detected in endothelial cells upon EV transfer, and only when a sequence-specific probe is used for detection (green spots; single-molecule RNA FISH; maximum intensity merges of Z-stacks). *RNU6*, positive control; scramble, negative control. Blue, nuclei (DAPI); scale bar, 10 μm. Cell membrane area is outlined by white dashed lines. *P* versus control (Student's *t*-test. (**d**) EV uptake does not induce miRNA expression in BMEC. BMEC were incubated with α-amanitin before EV uptake. miRNAs (miR-451a and miR-126) and VCAM-1 were quantified by qRT-PCR in endothelial cells upon EV incubation. qRT-PCR results from all experiments are normalized by the 2^-Ct^ method, using *RNU6* as a reference and expressed as mean and fold induction over control (mean ± s.e.m.; *n*=3 experiments), *P* versus control (Student's *t*-test).

Given the efficient uptake and perinuclear localization of EVs in endothelial cells, we hypothesized that they may deliver regulatory miRNAs to the endothelial cell. For the following experiments we focused on miR-451a because this is the most abundant miRNA species in EVs, and its expression is restricted to cells of the erythropoietic lineage, while not being expressed in other cell types including endothelial cells^38^,^39^. First we determined miR-451a levels in endothelial cells by qRT-PCR upon EV internalization, demonstrating dose-dependent miRNA accumulation of up to 50-fold above background (Fig. 6a). Detection of miRNA in BMEC is resistant to RNAse 1 and trypsin treatment, demonstrating that the detected miR-451a signal originates from internalized EVs rather than from contaminating serum components or EVs on the BMEC surface (Fig. 6b). We were also able to directly detect and quantify miR-451a copy number in endothelial cells upon EV uptake by RNA fluorescent *in situ* hybridization, while a scrambled probe or endothelial cells without added EVs show only background signal (Fig. 6c).

To exclude the possibility that miR-451a expression is induced in endothelial cells upon EV uptake, rather than transferred with EVs, we treated BMEC with α-amanitin before incubation with EVs. miRNAs are transcribed by RNA polymerase II, which can be efficiently inhibited by the fungal metabolite α-amanitin^40^. The increase in miR-451a expression upon EV treatment was not affected by α-amanitin, whereas levels of the endogenous control miRNA miR-126 remained constant (Fig. 6d). In contrast induced expression of VCAM-1 upon EV uptake was inhibited by α-amanitin. Altogether these experiments demonstrate that miR-451a is transferred to endothelial cells via EVs, rather than expressed in endothelial cells.

### EVs alter gene expression and barrier properties in endothelial cells

**Figure 7:**
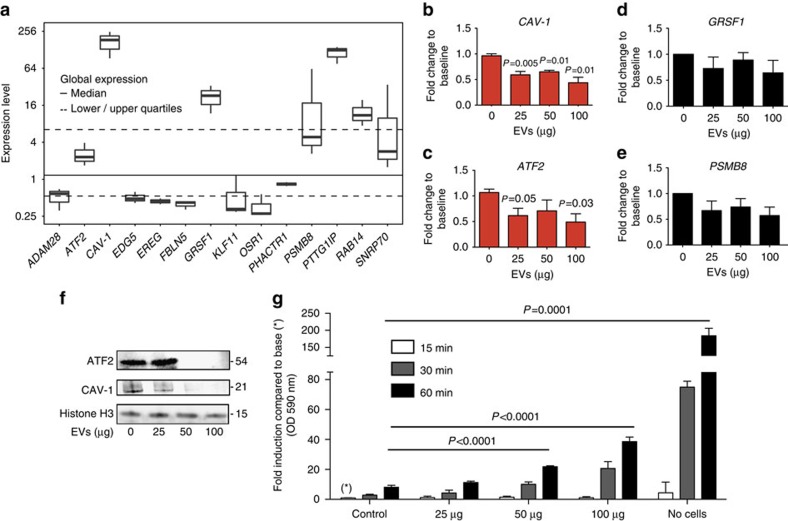
Downregulation of miR-451a target genes in endothelial cells upon EV uptake. (**a**) Enodogenous expression of miR-451a targets in endothelial cells. Shown is a boxplot for gene expression distribution of putative and known miR-451a targets in untreated BMEC cells^41^, that is, only the control samples from GEO platform GPL2986 (three in total) are included in the analysis. Most variable probe sets were used to represent gene expression when mapping from probe sets to genes isn't unique. Note the y-axis is log2 scaled. (**b**–**e**) Expression of miR-451a targets upon EV incubation. Potential miR-451a target transcripts were quantified by qRT-PCR. Two target genes, (**b**) *CAV-1* and (**c**) *ATF2* show significant downregulation, while *GRSF1* (**d**) and *PSMB8* (**e**) are not affected significantly in its transcript level. Results are normalized by the 2^-Ct^ method using *EF-1*, and with 18 S rRNA as a reference and expressed as mean (±s.e.m.) fold induction over untreated control (*n*=3 experiments), *P* versus control (Student's *t*-test). (**f**) Western blot analysis of ATF2 and CAV-1 demonstrates depletion of protein for both markers below detection levels upon incubation with 50 and 100 μg of EVs. (**g**) Effect of EV uptake on endothelial barrier. Endothelial cells were incubated with an EV titration and rhodamine-labelled dextran to measure permeability across a trans-well membrane over time. Permeability across the endothelial layer is significantly increased after 2 h of incubation with 50 and 100 μg of EVs. Data represent mean (±s.e.m.) from 3 experiments, *P* versus control (Student's *t*-test).

Next we tested whether internalized EVs can target endothelial gene expression through RNA interference of EV-derived miRNA complexes on target genes. miR-451a target gene prediction using available search tools (Targetscan, MicroSom, Microrna, DIANA lab) suggested multiple possible targets (Supplementary Table 1), either within coding regions of genes (that is, with a 100% match for endonucleolytic cleavage) or within the 3′-UTR of genes (that is, with a partial match for subsequent mRNA degradation). We confirmed expression of a subset of these putative targets in the BMEC line by microarray expression analysis^41^(Fig. 7a). To evaluate possible expression regulation by EV-derived miRNAs in BMEC, we focused on the four previously validated miR-451a targets, *CAV-1* (refs 42, 43), *ATF2* (ref. 44), *GRSF1* (ref. 45) and *PSMB8* (ref. 46), all of which are expressed above the median of all genes in this BMEC line (Fig. 7a). Specifically, we quantified transcript levels by qRT-PCR in endothelial cells upon EV incubation compared with control. Indeed 2 markers, *CAV-1* and *ATF2*, both with partial miR-451a target sites in their 3′-UTR (Supplementary Fig. 2), showed significant downregulation after 24 h of EV incubation compared with untreated control cells (Fig. 7b,c) and similar in magnitude to α-aminitin treatment (Supplementary Fig. 3). In contrast *GRSF1* (Fig. 7d) and *PSMB8* (Fig. 7e) were not affected.

*CAV-1* encodes Caveolin-1, a scaffolding protein and main component of caveolae, specialized lipid rafts in the plasma membrane of many vertebrate cell types with a role in signal transduction and endocytosis^47^. *ATF2* encodes Activating Transcription Factor 2, a multifunctional transcription factor present in various cell types including endothelial cells^48^. Western blot analysis demonstrated that both CAV-1 and ATF2 were also downregulated on a protein level after incubation with EVs (Fig. 7f).

As CAV-1 is known to regulate expression of proteins required for barrier function in the microvascular endothelium^49^,^50^ we wanted to test whether EV uptake showed altered barrier function compared with control cells. For this purpose, we investigated the integrity of the endothelial barrier using an *in vitro* permeability assay. In this assay, we measured the effect of EV uptake on traversing of rhodamine-labelled dextran diffusion through BMEC monolayers grown on 0.4 μm *trans*-wells filters. This experiment demonstrated dose- and time-dependent permeability increase upon EV incubation compared with untreated control cells (Fig. 7g). In summary, these experiments provided the first evidence of a link between EV uptake and altered endothelial function via changes in miR-451a target gene expression.

### miR-451a alters endothelial cell function

**Figure 8:**
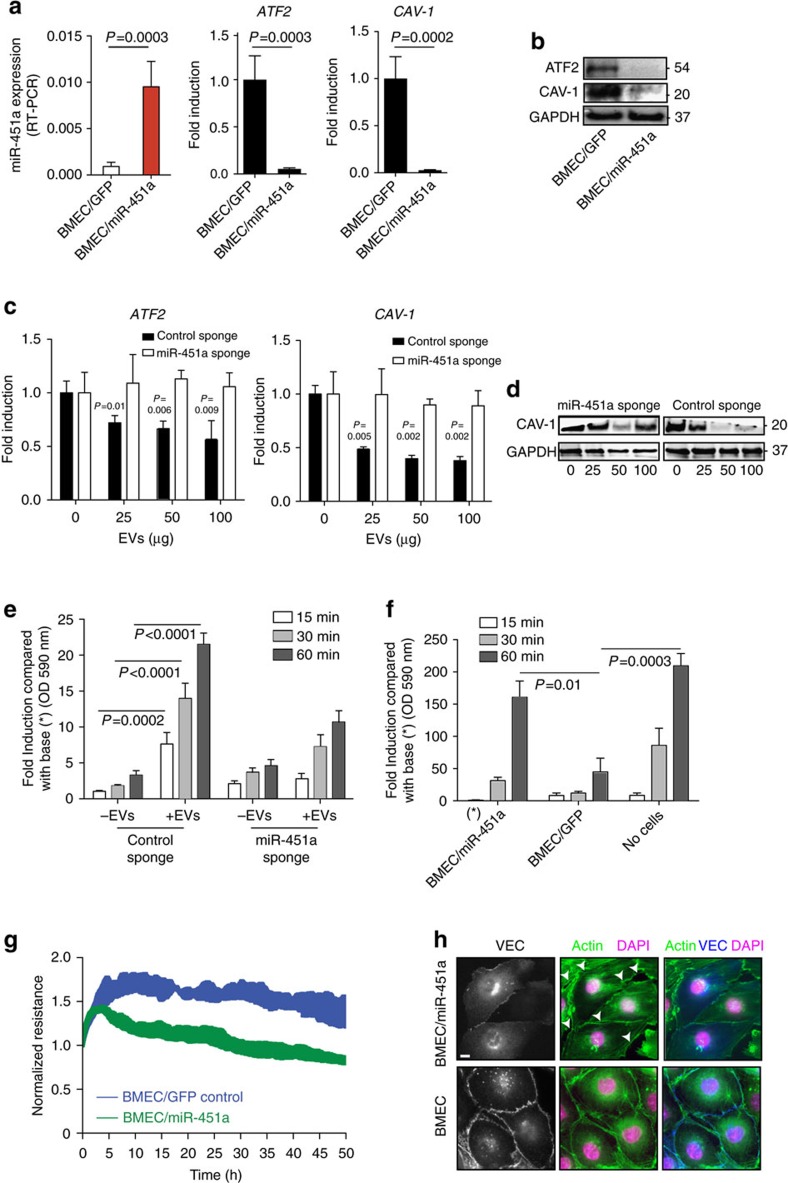
miR-451a alters endothelial barrier properties. (**a**,**b**) Effect of miR-451a over-expression. Endothelial cells were transduced with a lentiviral vector expressing miR-451a (BMEC/miR-451a) or GFP control (BMEC/GFP). Expression of *CAV-1* and *ATF2* was assessed on a transcript level by qRT-PCR (**a**) and on a protein level by western blot (**b**). At both levels the two markers are significantly reduced in BMEC/miR-451a compared with GFP control and wild-type BMEC. qRT-PCR results are normalized by the 2^-Ct^ method using *GAPDH*, and expressed as mean (±s.e.m.) (*n*=3 experiments), p versus control (Student's *t*-test). (**c**,**d**) miRNA inhibition via miRNA Sponge. Endothelial cells were transduced with miR-451a Sponge miRNA or control Sponge miRNA before addition of EVs and target gene and protein expression in endothelial cells was determined by qRT-PCR (**c**) and western blot (**d**). Expression of both target genes, *CAV-1* and *ATF2*, is restored compared with BMECs transduced with control Sponge. qRT-PCR results are normalized by the 2^-Ct^ method using *EF-1*, and with 18 S rRNA as a reference and expressed as mean (±s.e.m.) fold induction over untreated control (*n*=3 experiments), *P* versus control (Student's *t*-test). (**e**) Permeability of BMECs transduced with miR-451a Sponge or control Sponge was assessed by measuring the appearance of rhodamine-labelled dextran in the bottom well of a trans-well set up after a 1 hour time-course. The absorbance at 15, 30 and 60 min time points was compared with the control Sponge condition. Data represent mean (±s.e.m., *n*=6), *P* versus control (Student's *t*-test). (**f**,**g**) Altered barrier properties in BMEC/miR-451a line. Permeability assays using rhodamine-labeled dextran (**f**) and TEER assays (**g**) were performed over time, with BMEC/miR-451a and the BMEC/GFP control line. Both experimental approaches demonstrate significant reduction in endothelial barrier properties in BMEC/miR-451a compared with control. (**h**) BMEC control and BMEC/miR-451a cells were plated for 36 h before fixation, permeabilization and staining for actin and the junctional protein VE-cadherin (VEC). White arrows indicate presence of gaps in the BMEC/miR-451a line. Scale bar, 10 μm. TEER, trans-endothelial electrical resistance.

To ascertain that *CAV-1* and *ATF2* downregulation is indeed mediated by EV-derived miR-451a, we used two complementary approaches to directly alter miR-451a expression in BMEC and measure the effect on target gene expression. First we transduced BMEC with a lentiviral expression vector containing miR-451a (or GFP control). In the resulting BMEC/miR-451a line miR-451a was greatly induced while *CAV-1* and *ATF2* expression was reduced both on transcript and protein level, when compared with the GFP control (BMEC/GFP) and BMEC (Fig. 8a,b). In a second experiment, we transiently expressed Sponge miR-451a in endothelial cells before incubation with EVs. Sponge miRNA binds and sequesters cellular target miRNAs and thereby neutralizes them. Indeed expression of Sponge miR-451a but not of a control Sponge miRNA restored CAV-1 and ATF2 expression on mRNA and protein levels, confirming that they are genuine targets of EV-derived miR-451a (Fig. 8d). Furthermore the overexpression of a miR-451a sponge preserved the permeability of the endothelial cell barrier properties upon EV treatment (Fig. 8e). Together these experiments directly demonstrate that *CAV-1* and *ATF2* are miR-451a targets. Importantly, we were also able to recapitulate the observed effect of EVs on endothelial barrier properties in the BMEC/miR-451a line but not in the BMEC/GFP control by permeability assays measuring rhodamine-labelled dextran accumulation (Fig. 8f). Growth curves indicated a significant reduction in trans-endothelial electrical resistance in BMEC/miR-451a compared with BMEC/GFP (Fig. 8g), further confirming the role of miR-451a in regulation of endothelial barrier formation. In addition, miscoscopy experiments performed 36 h after cell plating showed decreased levels of cortical actin, increased stress fibre formation, discontinuous junctional VE-Cadherin and presence of gaps between cells in the BMEC/miR-451a cell line compared with BMEC, demonstrating that induction of miR-451a leads to vascular alteration (Fig. 8h). Of note, BMEC/miR-451a cells do not seem to reach overall confluence (intact monolayer) at any time point. Altogether these experiments confirmed a regulatory function for EV-derived miRNA-complexes in endothelial gene expression and barrier function.

## Discussion

We have recently demonstrated that EVs derived from iRBCs are transferred between iRBCs and capable of promoting the switch to formation of transmission stages^11^. We have also demonstrated that they are efficiently internalized by macrophages and induce a strong inflammatory response^11^. Here we show that EVs derived from iRBCs contain host miRNAs, derived from the mature RBCs, which form a functional RISC complex with Ago2. Importantly we show that this RISC complex is capable of specifically silencing gene expression in endothelial cells and alter their barrier properties. Altogether, these findings provide a mechanistic link between malaria infection and vascular dysfunction.

In our previous study, we demonstrated that EVs derived from iRBCs contain small RNA species^11^, in line with observations of EV-mediated transfer of RNA including miRNA in many eukaryotic cell types^51^,^52^. Profiling of human miRNA species revealed presence of a small set of miRNAs in EVs, with miR-451a and let-7b having the highest expression level. These two miRNAs are abundant in cells of the eyrthroid lineage including mature RBCs^23^. miRNAs are also present in human serum in a stable complex with lipids^17^,^29^. For example, miR-223 can form a complex with high-density lipoprotein, and this complex is efficiently transferred to co-cultured hepatocytes where it regulates transcription of miR-223 target genes^17^.

Mature RBCs, or normocytes, are terminally differentiated cells without nucleus and organelles. Erythropoiesis is a complex process during which hematopoietic stem cells undergo a series of differentiation steps and ultimately enucleate to give rise to reticulocytes, which are released from bone marrow into the blood circulation and mature rapidly into the terminal normocytes. Several miRNA species have an essential role during erythropoiesis and RBC maturation. For example, miR-320 is expressed in reticulocytes and essential for the downregulation of CD71 (ref. 23). Mice deficient in miR-451a show a reduction in haematocrit, which is caused partly by an increased susceptibility of RBCs to oxidative damage^24^.

We demonstrate that RBCs progressively lose the RNA interference machinery during their maturation, while retaining Ago2. Specifically, we detected expression of DICER and Drosha in erythroid cells until day 18, whereas Ago2 was still detectable in mature RBCs. Using confocal microscopy we demonstrated that Ago2 localizes to the cell periphery and disappears during the late iRBC cycle. We previously described that EVs are secreted during the later stages of parasite maturation, which coincides with degradation of the host cell cytoskeleton, and increase of cytoplasmic Calcium levels^11^. The observed dynamics of Ago2 localization in iRBCs and direct evidence for its localization in EVs therefore strongly suggest that Ago2 is quantitatively released by EVs. Importantly, we also demonstrate that specific miRNA species, notably miR-451a and let-7b, are associated with Ago2 in EVs and that this complex is active. We demonstrate both endonucleolytic activity through cleavage of specific target probes, and 3′-UTR association and subsequent target gene degradation in HEK293 cells incubated with EVs derived from iRBCs. This is the first demonstration of a functional RISC complex in mature RBCs. Recent studies have demonstrated Dicer-independent processing of miR-451a directly through Ago2 (refs 53, 54), providing a possible explanation for the presence of processed miR-451a in mature RBCs.

Multiple studies have demonstrated transfer of functional miRNAs via EVs to target cells. Endothelial cells are targeted by many EVs secreted by blood cells. For example miR-150 is selectively packaged into macrophage-derived EVs and transferred to endothelial cells in which it suppresses *c-Myb* expression and enhances cell migration^55^. Under hypoxic conditions, myeloma cells produce exosomes containing miR-135b that suppresses *hypoxia-inducible factor 1* in endothelial cells, thereby enhancing endothelial tube formation to control angiogenesis^56^. Also, HDL-bound miR-223 suppresses ICAM-1 expression in endothelial cells^57^. Our studies revealed that EVs derived from iRBCs can regulate gene expression in the recipient endothelial cells. Specifically, we demonstrate that EV-derived miR-451a targets expression of the endothelial cell markers *ATF2* and *CAV-1*. It has previously been shown that miR-451a inhibits *ATF2* expression by binding to its 3′-UTR^44^. ATF2 is a cAMP-response element-binding protein with a basic leucine zipper (bZIP) domain, through which it interacts with other bZIP proteins^44^. ATF2 induces a variety of genes that are involved in anti-apoptotic signalling^58^, and its inhibition by siRNA induces apoptosis in a melanoma model^59^. Downregulation of ATF2 by miR-451a might therefore contribute to the observed apoptosis of endothelial cells during malarial infection^60^. The second marker that is a target of EV-derived miR-451a in endothelial cells is Caveolin-1 (CAV-1). CAV-1 is the principal component of caveolae, distinct lipid- and cholesterol-rich invaginations at the plasma membrane that function as regulators of signal transduction. *CAV-1* expression is regulated by several miRNAs, including miR-451a (refs 42, 43). CAV-1 regulates expression of proteins required for barrier function in the microvascular endothelium: silencing of *CAV-1* by siRNA leads to reduction of the junction-associated proteins Zona occludens protein 1 (ZO-1) and occludin expression, as well as the adherence-related proteins VE-cadherin and b-catenin^49^. Similarly, *CAV-1* knock-out mice exhibit a vascular hyperpermeability phenotype^61^. In line with these observations, we demonstrate that ectopic expression of miR-451a in the BMEC line results in reduced barrier properties and redistribution of tight junction markers.

Collectively, our data obtained through a multifaceted *in vitro* approach strongly support a model where EV release contributes both to local and systemic production of pro-inflammatory cytokines and chemokines and to vascular dysfunction, promoting endothelial activation, leakage and parasite sequestration as well as pathology during malaria infection. Recent observations that severe malaria is associated with a dramatic increase in EVs in the plasma of malaria patients^14^ strongly support a role *in vivo* for EVs in contributing to pathology. We are currently validating this system in an *in vivo* model in order to determine its implications under physiological conditions.

## Methods

### *Plasmodium falciparum in vitro* culture

Parasites of the *P. falciparum* reference strain 3D7 were used for this study. Parasites were kept in fresh type 0+ human erythrocytes (Research Blood Components, Boston, MA), suspended at 2% hematocrit in HEPES-buffered RPMI 1640 containing 10% (w/v) heat inactivated human serum, 0.5 ml gentamycin, 2.01 g sodium bicarbonate and 0.05 g hypoxanthine at pH 6.74. The complete medium was depleted from EVs and debris by ultracentrifugation at 100,000*g* for 1 h. Cultures were kept in a controlled environment at 37 °C in a gassed chamber at 5% CO_2_ and 1% O_2_.

### Isolation of extracellular vesicles

EVs from infected RBCs were isolated from cell culture supernatants as described^11^. To remove cells and debris, cell suspensions were sequentially pelleted at 600, 1,600, 3,600 and finally 10,000*g* for 15 min each. To further concentrate the EV population, the filtrate was passed through a Vivacell 100 filter (100 kDa molecular weight cutoff; Sartorius, Bohemia, NY). The concentrated supernatant was then pelleted at 100,000*g*, the pellet resuspended in PBS and layered on top of a 60% sucrose cushion and spun at 100,000*g* for 16 h. The interphase was collected and washed with PBS twice at 100,000*g* for 1 h.

### Maturation of human RBCs from hematopoietic stem cells

Generation of *in vitro* matured RBCs was conducted essentially as described previously^27^. Bone marrow-derived CD34+ hematopoietic stem cells (Lonza, Switzerland) were grown in Iscove's Modified Dulbecco-based medium (Biochrom, Germany) supplemented with 4 mM glutamine (Sigma, St Louis, MO), 330 μg ml^−1^ holo-transferrin (BBI solutions, UK), 10 μg ml^−1^ insulin (Sigma), 2 IU ml^−1^ heparin sodium salt (Affymetrix, Santa Clara, CA), Pen Strep (Thermo-Fisher, Grand Island, NY), 100 ng ml^−1^ stem cell factor (SCF)(R&D Systems, Minneapolis, MN), 10^−6^ M hydrocortisone (Sigma), 5 ng ml^−1^ IL-3 (R&D Systems), 3 U ml^−1^ erythropoietin (Amgen, Cambridge, MA) and 5% pooled solvent/detergent treated human plasma (Octaplas, Octapharma, Switzerland). Hydrocortisone and IL-3 were removed from the culture on day 8, SCF on day 11.

### Fractionation of infected red blood cells

Synchronized ring stage (12 h post invasion) parasites were enriched to >95% parasitemia by an initial Streptolysin O treatment of 2.5 haemolytic units (HU) for 20 min (ref. 62). Later stages were enriched using magnetic separation with a MACS CS column (Miltenyi Biotec, Cambridge, MA). Purified iRBCs were then fractionated by sequential treatment with tetanolysin (1 HU for 20 min) and saponin (0.035% for 5 min).

### RNA detection by Bioanalyzer, Nanostring and Northern blot

Total RNA was isolated from EVs, iRBCs and RBCs using miRNeasy kit (Qiagen, Valencia, CA). The concentration and integrity of total RNA was measured using a NanoDrop-1000 spectrophotometer (Thermo Scientific, Wilmington, DE). Size distribution was determined by Agilent 2100 Bioanalyzer with the Agilent Small RNA Chip (Agilent Technologies, Santa Clara, CA).

For Nanostring expression analysis total RNA samples were processed according to manufacturer's instructions for the nCounter Human miRNA Expression Assay kit (NanoString, Seattle, WA). Briefly, 100 ng of total RNA from each sample was used as input for the nCounter Human miRNA preparation. Hybridization was conducted for 16 h at 65 °C. Subsequently, the strip tubes were placed into the nCounter Prep Station for automated sample purification and reporter capture. Abundance of specific target molecules was quantified on the nCounter Digital Analyzer by counting the individual fluorescent barcodes and assessing the target molecules. Data was extracted using the nCounter RCC Collector.

To detect miRNA by northern blot 200–500 ng of total RNA per sample was loaded onto a 15% TBE-Urea gel (Thermo-Fisher), run and transferred to a Nylon neutral Hybond-NX membrane (GE Healthcare, Wilmington, MA) for 90 min at 15 V in a semi-dry electrophoretic transfer cell unit (Bio-Rad, Hercules, CA). Membranes were ultraviolet-crosslinked. Blots were pre-hybridized at 37 °C for at least 30 min in ULTRAhyb solution (Ambion). Blots were then hybridized overnight with a DIG labelled specific locked-nucleic acid probes (Exiqon, Woburn, MA) for hsa-miR-451a, hsa-miR-106b and let-7b, at a final concentration of 0.5 nM in ULTRAhyp at 37 °C. Chemiluminescence detection was performed following incubation with a horseradish peroxidase-conjugated antibody against DIG. Northern blots were performed using RNAs from three separate experiments.

### Western blot and immunofluorescence analysis (IFA)

Samples were collected and purified as described in each specific experiment. For SDS–polyacrylamide gel electrophoresis the pellet was washed three times in PBS and taken up in reducing SDS sample buffer (Invitrogen, Carlsbad, CA). Proteins were separated on 4–12% Bis-Tris gels (Invitrogen) and proteins transferred onto Immun-Blot PVDF membranes (Biorad), according to standard protocols. Antibodies used are anti-Ago2/EIF2C2 (clone 2E12-1C9, Abnova), anti-Dicer1 (clone 13D6, Abcam), anti-Drosha (Abcam), anti-stomatin (clone M-14; Santa Cruz Biotechnologies) and anti-Histone H3 (Abcam). Secondary antibodies (IR-Dye-conjugated) were goat anti-rabbit and goat anti-mouse immunoglobulin (LICOR, Lincoln, NE). Immunoreactive bands were detected using the Odyssey imaging system (LICOR).

For IFA, iRBCs and RBCs were fixed in 4% paraformaldehyde and 0.0075% glutaraldehyde for 30 min. Cells were incubated with anti-Ago2/EIF2C2 (clone 2E12-1C9, Abnova) 1:50 for 2 h followed by Alexa Fluor 488 secondary IgG antibodies (1:1,000; Molecular Probes, Eugene, OR) for 1 h. Nuclei were labelled with DAPI nuclear stain (Roche) at 0.2 mg ml in Vectashield mounting solution (Vector Labs, Burlingame, CA). Imaging was performed on an Olympus BX62 microscope fitted with a cooled Hamamatsu Orca AG camera. The microscope, filters, and camera were controlled using iVision version 4.0.9 software (BioVision, Milpitas, CA).

For flow cytometry, iRBCs and RBCs were fixed in 4% paraformaldehyde and 0.0075% glutaraldehyde for 30 min. Cells were incubated with anti-Ago2/EIF2C2 (clone 2E12-1C9, Abnova) 1:50 for 2 h followed by Alexa Fluor 594 secondary IgG antibodies (1:1,000; Molecular Probes) for 1 h. Nuclei were labelled with Sybr Green (Life Technologies, Carlsbad, CA).

Cytometry data were collected with a MACSQuant VYB flow cytometer.

### Immuno electron microscopy of EVs

Purified EVs from *P. falciparum* cultures cells were fixed with 4% paraformaldehyde in 0.1 M sodium phosphate buffer at pH 7.4 for 2 h at room temperature. Before freezing in liquid nitrogen cell pellets were infiltrated with 2.3 M sucrose in PBS containing 0.2 M glycine for 15 min. Frozen samples were sectioned at −120 °C and 60–80 nm sections were transferred to formvar-carbon coated copper grids. Gold labelling was carried out at room temperature on a piece of para film. For Ago2 labelling, antibodies were diluted in 1% bovine serum albumin (BSA) in PBS. Grids were floated on drops of 1% BSA for 10 min to block for unspecific labelling, transferred to 5 μl drops of anti-Ago2 gold 10 nm for 30 min, then washed in four drops of PBS and six drops of double distilled water. The sections were contrasted and embedded by floating the grids on a mixture of 0.3% uranyl acetete in 2% methyl cellulose for 5 min. Excess liquid was blotted off on a filter paper and the grids were examined in a JEOL 1200EX Transmission electron microscope (JEOL, Peabody, MA) and images were recorded with an AMT 2k charge-coupled device camera (AMT Woburn, MA).

### Size exclusion chromatography

Experiments to determine possible presence of serum-derived Ago2-miRNA complexes were performed essentially as described by Arroyo *et al*.^29^

Briefly, sephacryl S-500 resin (GE Healthcare) was packed in a chromatography column (0.9 Å∼ 30 cm, 19.1 ml bed volume). Before injection, the column was equilibrated with 25 ml of PBS solution at 0.5 ml min^−1^ at room temperature. The column was injected with 0.5 ml of undiluted fresh serum or purified EVs and eluted at 4 °C for ∼1 h with PBS solution (pH 7.4) at a flow rate of 0.5 ml min^−1^. A total of 25 fractions of 1 ml each were collected. Fractions were stored at 4 °C before use. Protein molecular weight standards included BSA (67 kDa; GE Healthcare) and tyrosine (0.181 kDa; Sigma-Aldrich).

### Proteinase K Treatment of purified EVs

Recombinant, PCR-grade, DNase/RNase-free proteinase K (Roche, Switzerland) was prepared in RNase-free water. Two samples of EVs were prepared on ice. At 0 min, 0 mg ml^−1^ or 5 mg ml^−1^ proteinase K was added to the plasma, and aliquots were immediately removed and denatured in five volumes of QIAzol. The remainder of each sample was incubated at 55 °C. Aliquots were removed at time points indicated and denatured in QIAzol.

*Caenorhabditis elegans* spike-in oligonucleotides were added to denatured samples, and the samples were stored at −80 °C as they were generated. RNA was isolated as described above. Samples were also taken for SDS–polyacrylamide gel electrophoresis and western blot analysis.

### RISC assays

RISC assays were essentially performed as previously described^63^. Sensor RNA transcripts harbouring binding sites complementary to endogenous miRNAs hsa-miR-451a or hsa-let-7b were prepared by *in vitro* transcription using T7 RNA promoter/polymerase (T7 MEGAshortscript kit, Ambion). DNA templates were prepared by annealing of two complementary oligonucleotides. After gel purification, the RNA sensors were dephosphorylated with calf intestine alkaline phosphatase (Roche), 5′-end labelled with [γ-^32^P] ATP (Perkin-Elmer) using 10 U opti-kinase (USB). For activity assays we prepared protein extracts from EVs in S100 lysis buffer (40 mM Hepes, 100 mM potassium acetate, 5 mM MgCl2, 2 mM DTT, 0.35% (v/v) Triton X-100, pH 7.6). We incubated 50 μg of S100 protein extracts or Ago2 immunoprecipitates with the ^32^P-labelled RNA sensor (10,000 c.p.m.) in assay buffer containing 20 mM Hepes, 50 mM potassium acetate, 2.5 mM MgCl2, 1 mM ATP, 0.2 mM GTP, 1 mM DTT, 2.5% (v/v) Superase-In, 0.18% (v/v) Triton X-100, pH 7.6, at 30 °C for 90 min. Some experiments were performed with pre-incubation in the presence of proteinase K (1 mg ml^−1^) or EDTA (5 mM). The latter reaction was stopped by adding 0.5 mg ml^−1^ of proteinase K and incubation at 50 °C for 30 min. After a phenol/chloroform extraction step, the RNA products were precipitated, resuspended in water, separated by 10% polyacrylamide gel electrophoresis in 7 M urea (Life Technologies) and analysed by autoradiography.

### Reporter gene activity assays

Reporter gene activity assays were performed essentially as described previously^64^. A miR-451a reporter construct was created by inserting a sequence complementary to hsa-miR-451 into the Xho I and Not I sites of psiCHECK-2 vector (Promega, Madison, WI), downstream of the *Renilla luciferase* (*Rluc*) reporter gene. *Firefly luciferase* (*Fluc*) was used as a normalization control. Constructs were transfected into HEK293 cells 24 h before incubation with EVs for up to 48 h. Rluc and Fluc activities were measured with Dual Glo-luciferase reagents (Promega) using a luminometer (Dynex Technologies, Chantilly, VA).

### Immunoprecipitation (IP) of Ago2-microRNA complexes

EVs derived from iRBCs were lysed in RNA IP lysis buffer (20 mM Tris-HCl pH 7.5, 150 mM NaCl, 1.5 mM MgCl_2_, and 0.25% NP-40), and the lysates cleared by centrifugation before IP using protein G-agarose beads (Roche) conjugated to an anti-Ago2 antibody (clone 2E12-1C9, Abnova) or isotypic IgG control (Novus), as described previously^63^. Ago2-associated miR-451a and let-7b was isolated by phenol/chloroform extraction and ethanol precipitation, reverse transcribed with the TaqMan MicroRNA reverse Transcription Kit (Applied Biosystems, Foster City, CA), and analysed by qRT-PCR using hsa-miR-451a or let-7b TaqMan Small RNA Assays (Applied Biosystems). The Ago2-miR-451a function was then evaluated in RISC activity assays, as previously described in the RISC activity section.

### Endothelial cell culture

A semi-immortalized human BMEC-1 (ref. 30) was cultured in DMEM (Life technologies) supplemented with 10% fetal bovine serum (Cedarlane, Burlington, Canada) and maintained at 37 °C under 5% CO_2_.

### miRNA Target predictions

The mature miR-451a sequence was obtained from miRBase ( http://www.mirbase.org). The human targets of HSA-miR451a were predicted using public Web-based prediction tools, including TargetScan ( http://www.targetscan.org), MicroCosm ( http://www.ebi.ac.uk/enright-srv/microcosm), microRNA.org ( http://www.microrna.org/microrna/getGeneForm.do) and Diana LAB ( http://diana.cslab.ece.ntua.gr/micro-CDS).

### RNA extraction and gene expression studies in BMEC-1

Total RNA was extracted from iRBC-derived EVs and BMEC-1 using TRIzol reagent (Invitrogen), and from the supernatant fraction using miRVana PARIS kit (Ambion). Reverse transcription reactions were performed with 1 μg total RNA using HiFlex miSCRIPT RTII kit (Qiagen) after DNase I treatment (Invitrogen). Mature miR-451a and selected mRNAs were detected by qRT-PCR using miScript Primer Assay kit and SYBR Green (Qiagen). Small nuclear RNA U6 (RNU6) (for miR-451a) and 18 S rRNA and EF1 (for mRNAs) were used as reference genes for relative quantitation using the 2^−Ct^ method.

### EV internalization assays with BMEC-1 and miRNA transduction

Purified EVs were labelled with PKH26 red fluorescent labelling kit (Sigma-Aldrich) and incubated with BMECs for 2, 6 or 12 h before washing cells three times to remove unbound EVs. For uptake inhibition experiments endothelial cells were co-incubated with different compounds, as described in Fig. 5. Cells were fixed, permeabilized and stained for F-actin with phalloidin-Alexa-Fluor-488 (Invitrogen) and with the nuclear dye Topro-3 (Thermo-Fisher). Epifluorescence was performed on a Axiovert 200M microscope (Carl Zeiss. Germany) equipped with a camera (Orca charge-coupled device; Hamamatsu Photonics, Japan) using a × 40 Plan-Neofluar (NA 1.3) oil immersion objective. Intensity analysis was performed with the Axiovision 4.6.3 software. Background subtraction was performed for each field. Confocal microscopy was performed on a Zeiss LSM 510 Laser Scanning Microscope using a × 63 Plan-Neofluar water-immersion objective.

### RNA Fluorescence *in situ* hybridization

We performed RNA fluorescent *in situ* hybridization staining essentially as described^65^, using 10 nM of DIG-labelled miRNA LNA probes (Exiqon). A scrambled miRNA LNA probe was used as a negative control. After a series of post-hybridization washes, the LNA signal was amplified using the Tyramide Signal Amplification PLUS Fluorescein Kit (Perkin-Elmer, Waltham MA) according to the manufacturer's instructions.

### Generation of BMEC/miR-451a and BMEC/GFP lines

For the construction of the miR-451a lentiviral vector, fragments containing the human miR-451a genomic clusters were amplified by PCR from human genomic DNA and directionally cloned into the *AgeI* and *EcoRI* sites of the pLKO-1 vector (Addgene, Cambridge, MA) using the primers: forward 5′- AAAAACCGGTCTAGTCCGGGCACCCCCAG -3′ and reverse 5′- AAAAGAATTCCCTACCCCCAATCCCACGC -3′. Mission miRNA inhibitor cel-miR-243-3p (Control Sponge) and hsa-miR-451a (Sponge) were purchased from Sigma-Aldrich.

Lentiviral particles were produced by transfecting HEK293T cells using the calcium phosphate method with packaging plasmid psPAX2 and envelope plasmid pMD2.G (both Addgene) and harvesting supernatants after 48 h, essentially as described by Alves *et al*.^66^ Viral supernatant was harvested 48 h post transfection, filtered (0.45 μm pore size), and transduced into BMEC cells in the presence of 8 μg ml^−1^ of polybrene (Sigma-Aldrich). After 24 h, cells were maintained in medium containing 0.6 μg ml^−1^ puromycin (Sigma-Aldrich) for selection.

### IFAs with wild-type and transduced BMEC lines

To investigate barrier function control BMECs and the transduced BMEC/miR-451a cell line were grown to confluence for 36 h before fixation, staining for VE-cadherin followed by AlexaFluor-conjugated secondary antibody, permeabilization and staining for phalloidin-Alexa-Fluor-488 to detect F-actin and DAPI to detect nuclei. Cells were analysed by epifluorescence as above.

### Permeability and TEER assays

The permeability of treated BMEC monolayers grown on transwell filters (0.4 μm pore size; Corning, Durham, NC) was assessed by the passage of rhodamine B isothiocyanate-dextran (average MW ∼70,000; Sigma). Briefly, rhodamine-dextran was added to the top well at 20 mg ml^−1^, and the appearance of fluorescence in the bottom well was monitored by measuring 40 μl medium aliquots in a time course using a multilabel microplate reader (Perkin-Elmer) at 544 nm excitation and 590 nm emission. The cells were incubated at 37 °C with 5% CO_2_.

For trans-endothelial electrical resistance assays endothelial cells were plated on gold-coated electrodes (40,000 cells per well) and trans-endothelial electrical resistance was monitored in real time by electric cell-substrate impedance sensing (Applied BioPhysics), as previously described^67^. Resistance (Ω) values were analysed by normalizing against baseline levels.

### Data availability

All relevant data are available from the authors. Also please note that all western blots shown in the manuscript have been cropped but full, uncropped blots are available in Supplementary Figs 4–6.

## Additional information

**How to cite this article**: Mantel, P.-Y. *et al*. Infected erythrocyte-derived extracellular vesicles alter vascular function via regulatory Ago2-miRNA complexes in malaria. *Nat. Commun.* 7:12727 doi: 10.1038/ncomms12727 (2016).

## Supplementary Material

Supplementary Information Supplementary Figures 1-6 and Supplementary Table 1

Supplementary Data 1 Nanostring miRNA expression analysis. Shown are normalized nanostring miRNA expression values for the 800 human miRNAs. Data are normalized based on the highest 100 miRNAs expressed.
