# Supplementary material for: Infected erythrocyte-derived extracellular vesicles alter vascular function via regulatory Ago2-miRNA complexes in malaria
Source: Nat Commun. 2016 Oct 10;7:12727. doi: 10.1038/ncomms12727 (PMC5062468; doi:10.1038/ncomms12727)
Supplement: Supplementary Information — Supplementary Figures 1-6 and Supplementary Table 1 [file ncomms12727-s1.pdf]

Supplementary Fig. 1

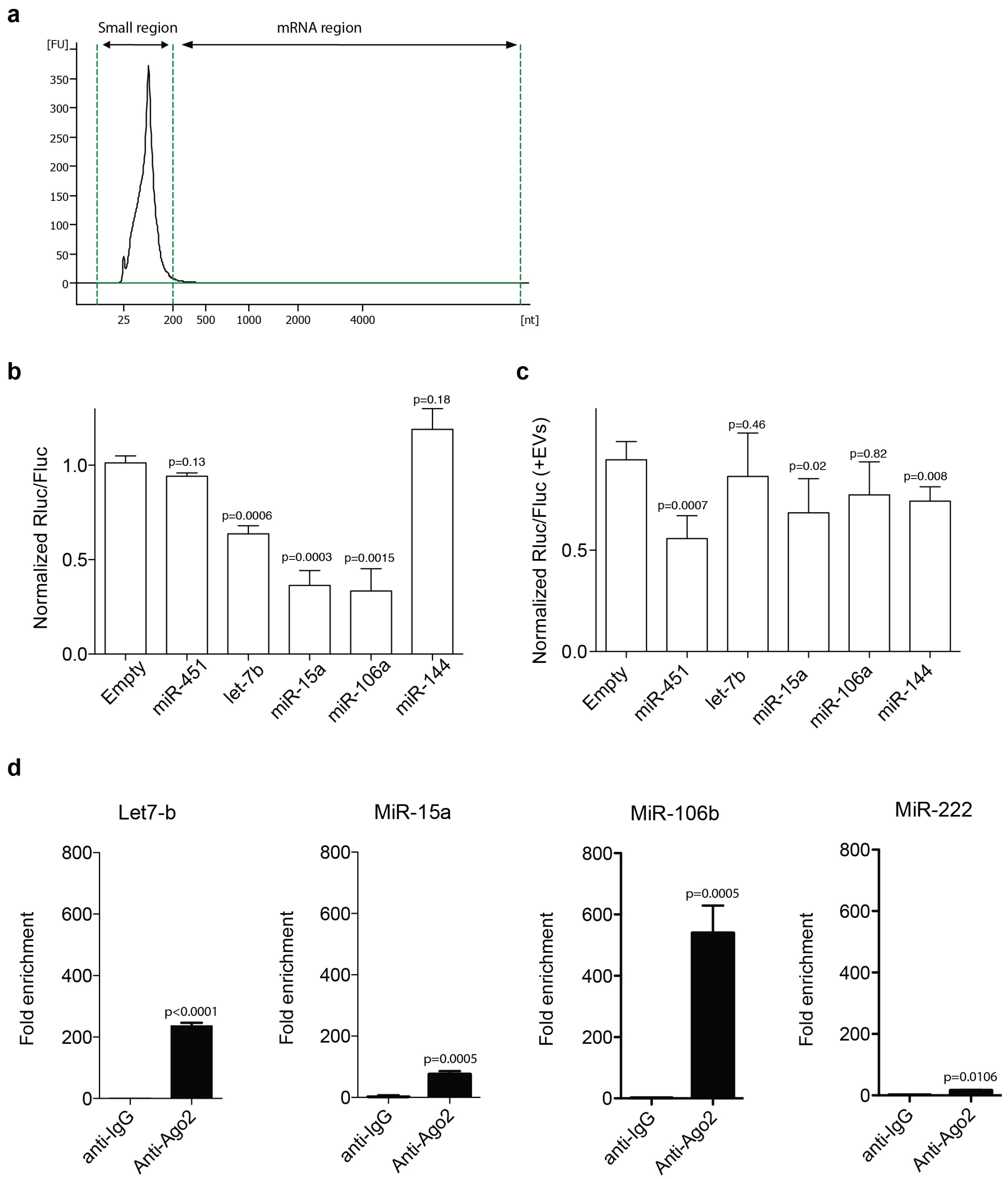

Supplementary Figure 1. Characterization of a functional Ago2-miRNA complex in EVs. (a) EVs derived from iRBCs contain no RNA species other than small RNAs. RNA samples were prepared from EVs and analyzed by Agilent Bioanalyzer RNA Chip. Only small RNA species < 200 nucleotides are detected. FU=Fluorescence units. **Characterization of a functional Ago2-**

miRNA complex in EVs: 3'UTR degradation activity (b,c). HEK293 cells transiently expressing a Renilla luciferase reporter gene with binding site complementary to miR-451a, let-7b, miR-15a in the 3' untranslated region of the reporter cassette were incubated for 48 h prior to luciferase activity measurements, either without **(b)** or with EVs derived from iRBCs **(c)**. miR-451a reporter is only significantly reduced upon addition of EVs, while let-7b, miR-15a and miR-106a are also in the control suggesting endogenous activity of these miRNAs in HEK293 cells, as described ***(Hafner et al)***. Results are normalized based on constitutive Firefly luciferase activity, and expressed as mean (± SEM) percentage of control (n = 3 experiments). *p < 0.05; **p < 0.01; ***p < 0.005 versus baseline or control (Student's t test). **(d) Ago2 association of miRNAs.** Protein extracts derived from purified EVs were subjected to immuno-precipitation (IP) using anti-Ago2 antibody, followed by Let7-b, miR-15a, miR-106b, miR-222 quantification using qRT-PCR. Results are normalized by the 2^-Ct method, using *RNU6* as a reference and expressed as percentage of the input (mean +/- SEM; n=4 experiments).

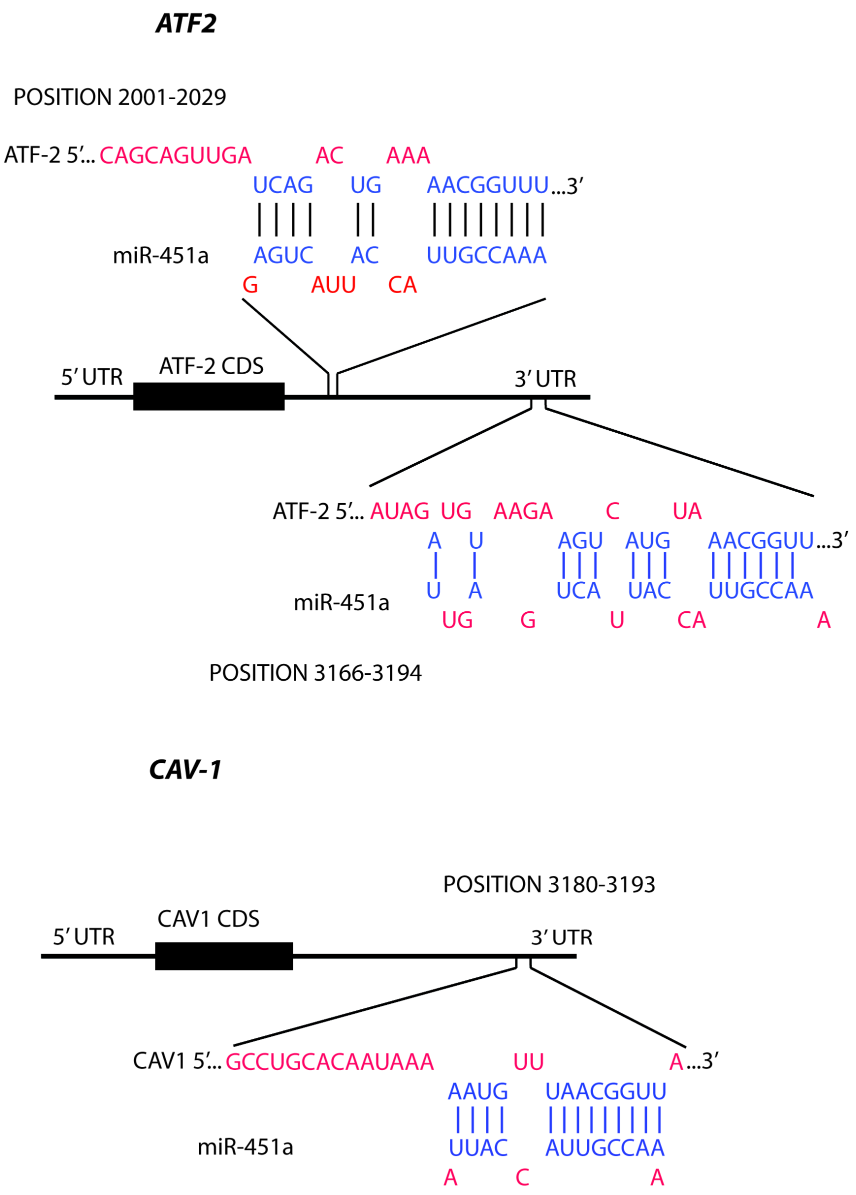

Supplementary Figure 2. Predicted miR-451a target sites in *CAV-1* and *ATF2*. Schematic representation of the 3'UTR of ATF2 (**a**) and CAV-1 (**b**). Predicted interactions of miR-451a with their binding sites are shown, with sequences of mature miR-451a aligned to these target sites highlighted.

Supplementary Fig. 3

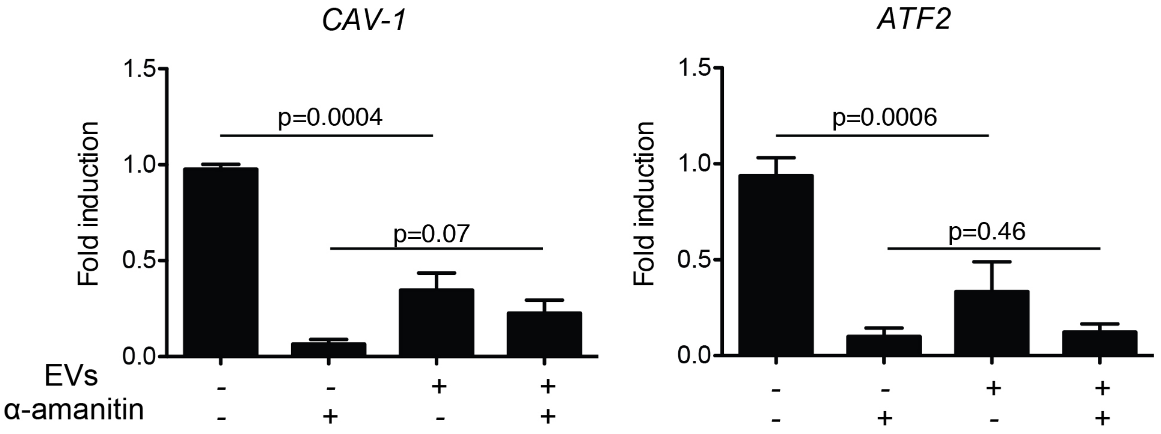

Supplementary Figure 3. Transcriptional dependence of *ATF2* and *CAV-1* expression levels in hBMECs. qRT- PCR analysis of *CAV-1* (left) and *ATF2* (right) levels in BMECs pre-incubated for 7 hours with α-amanitin, followed by incubation for 24 h with EVs (100 µg of total protein/ml). Relative changes normalized to 18 S rRNA presented as fold changes. Data represent the mean± SD (n = 4).

Supplementary Figure 4

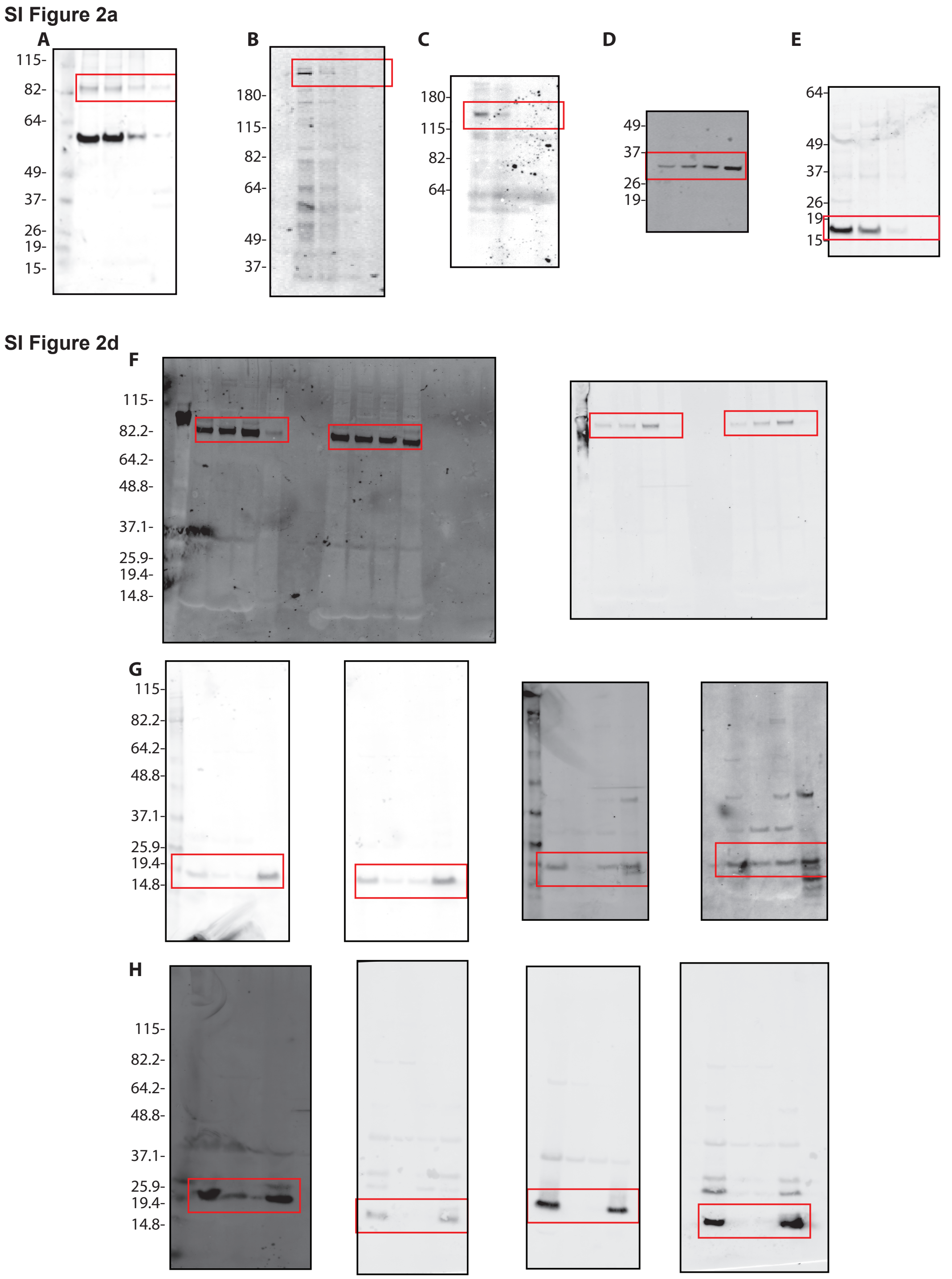

Supplementary Figure 4. Full, uncropped images of immunoblots shown in the main figure 2a and d. Molecular weight markers (BenchMark Pre-stained Protein Ladder, Thermofisher) are indicated on the left of the blots.

Supplementary Figure 5

SI Figure 3a

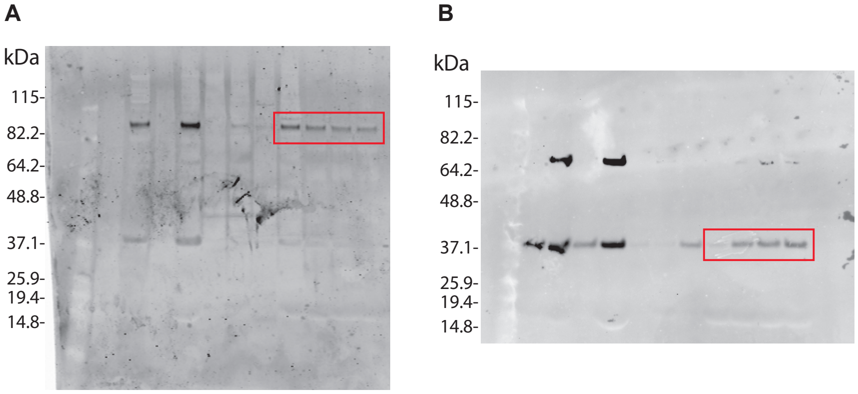

SI Figure 3e

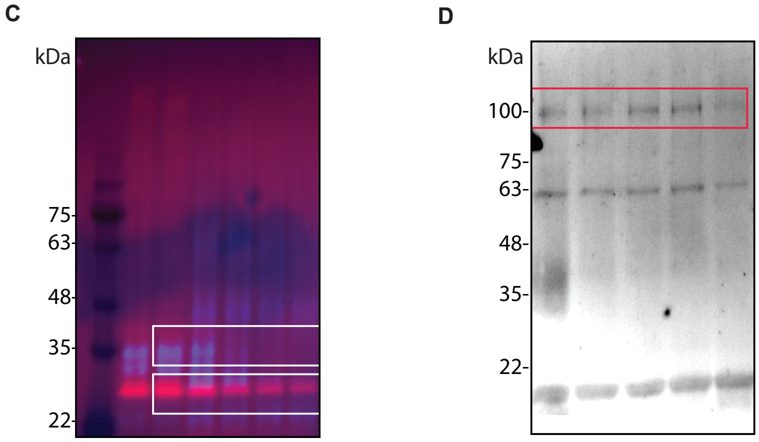

Supplementary Figure 5. Full, uncropped images of immunoblots shown in the main figure 3a and e. Molecular weight markers (BenchMark Pre-stained Protein Ladder, Thermofisher for A and B and Prestained Protein Ladder V, Geneaid for C and D) are indicated on the left of the blots.

Supplementary Figure 6

SI Figure 8b

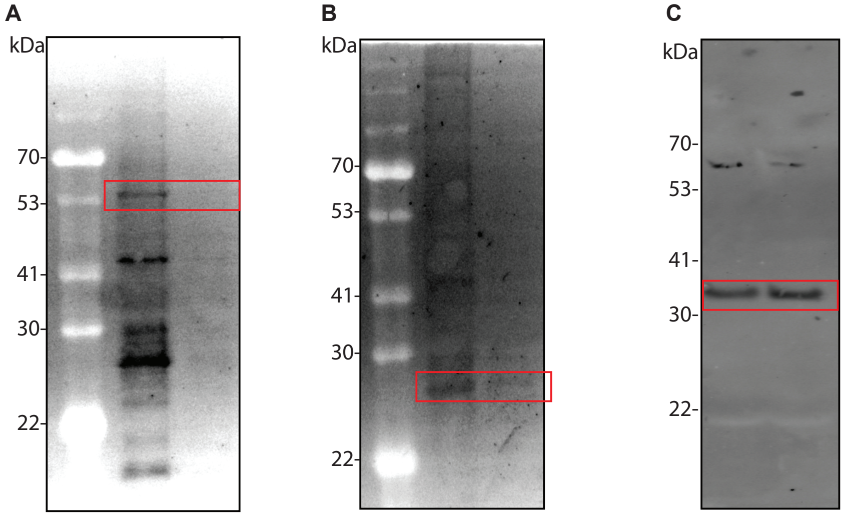

SI Figure 8d

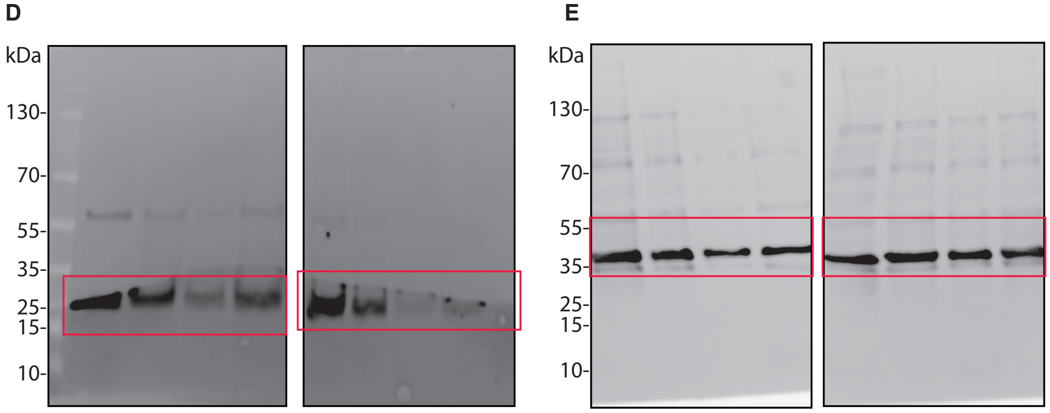

Supplementary Figure 6. Full, uncropped images of immunoblots shown in the main figure 8b and d. Molecular weight markers (Prestained Protein Ladder V, Geneaid) are indicated on the left of the blots.

Gene	RefSeq ID	Targetscan	Microscom	microrna.org	DIANA Lab
ADAM28	*NM_014265.4*	1			
ATF2	*NM_001880*		1	1	1
CAV-1	*NM_001172895.1*			1	1
EREG	*NM_001432.2*			1	
FBLN5	*NM_006329.3*		1	1	1
GRSF1	*NM_001098477.1*		2	2	3
KLF11	*NM_001177716.1*		1	1	
MST4	*NM_001042452.1*			1	
OSR	*NM_145260.2*	1	1	1	1
PHACTR1	*NM_001242648.1*				2
PSMB8	*NM_004159.4*	1	1	1	
PTTG1IP	*NM_001286822.1*			1	2
RAB14	*NM_016322.3*			2	2
S1PR2	*NM_004230.3*			1	1
SAMD4B	*NM_018028.2*		1	1	1
SNRNP70	*NM_001009820.1*				1

Supplemental Table 1. Predicted miR-451a target genes. Shown are targets based on 4 different prediction software packages: Targetscan, microcosm, microrna.org and DIANAlab.
